# Control of trichome formation in *Arabidopsis* by poplar single-repeat R3 MYB transcription factors

**DOI:** 10.3389/fpls.2014.00262

**Published:** 2014-06-10

**Authors:** Limei Zhou, Kaijie Zheng, Xiaoyu Wang, Hainan Tian, Xianling Wang, Shucai Wang

**Affiliations:** Key Laboratory of Molecular Epigenetics of Ministry of Education and Key Laboratory of Vegetation Ecology of Ministry of Education, Northeast Normal UniversityChangchun, China

**Keywords:** trichome formation, R3 MYBs, transcription factors, *Arabidopsis*, *Populus trichocarpa*

## Abstract

In *Arabidopsis*, trichome formation is regulated by the interplay of R3 MYBs and several others transcription factors including the WD40-repeat protein TRANSPARENT TESTA GLABRA1 (TTG1), the R2R3 MYB transcription factor GLABRA1 (GL1), the bHLH transcription factor GLABRA3 (GL3) or ENHANCER OF GLABRA3 (EGL3), and the homeodomain protein GLABRA2 (GL2). R3 MYBs including TRICHOMELESS1 (TCL1), TCL2, TRYPTICHON (TRY), CAPRICE (CPC), ENHANCER OF TRY AND CPC1 (ETC1), ETC2 and ETC3 negatively regulate trichome formation by competing with GL1 for binding GL3 or EGL3, thus blocking the formation of TTG1–GL3/EGL3–GL1, an activator complex required for the activation of the trichome positive regulator gene *GL2*. However, it is largely unknown if R3 MYBs in other plant species especially woody plants have similar functions. By BLASTing the *Populus trichocarpa* protein database using the entire amino acid sequence of TCL1, an *Arabidopsis* R3 MYB transcription factor, we identified a total of eight R3 MYB transcription factor genes in poplar, namely *P. trichocarpa TRICHOMELESS1* through *8* (*PtrTCL1–PtrTCL8*). The amino acid signature required for interacting with bHLH transcription factors and the amino acids required for cell-to-cell movement of R3 MYBs are not fully conserved in all PtrTCLs. When tested in *Arabidopsis* protoplasts, however, all PtrTCLs interacted with GL3. Expressing each of the eight *PtrTCL* genes in *Arabidopsis* resulted in either glabrous phenotypes or plants with reduced trichome numbers, and expression levels of *GL2* in all transgenic plants tested were greatly reduced. Expression of *PtrTCL1* under the control of *TCL1* native promoter almost completely complemented the mutant phenotype of *tcl*. In contrast, expression of *PtrTCL1* under the control of *TRY* native promoter in the *try* mutant, or under the control of *CPC* native promoter in the *cpc* mutant resulted in glabrous phenotypes, suggesting that PtrTCL1 functions similarly to TCL1, but not TRY and CPC.

## INTRODUCTION

Single-repeat R3 MYB transcription factors (R3 MYBs) are small proteins that typically contain ~100 amino acids, largely consist of a single MYB DNA-binding repeat, and are best characterized for their regulatory roles in trichome and root hair development (Wang and Chen, 2014). R3 MYBs are widely distributed in the plant kingdom, and they are encoded by a small subset of MYB transcription factor genes (Dubos et al., 2010). In *Arabidopsis*, there are a total of seven genes encoding R3 MYBs, including *TRYPTICHON* (*TRY*; Schnittger et al., 1999), *CAPRICE* (*CPC*; Wada et al., 1997), *TRICHOMELESS1* (*TCL1*; Wang et al., 2007), *TCL2*/*CAPRICE-LIKE MYB4* (*CPL4*; Gan et al., 2011; Tominaga-Wada and Nukumizu, 2012), *ENHANCER OF TRY AND CPC1* (*ETC1*; Esch et al., 2004; Kirik et al., 2004a), *ETC2* (Kirik et al., 2004b), and *ETC3*/*CPL3* (Simon et al., 2007; Tominaga et al., 2008). All seven R3 MYBs contain the residues [D/E]L×2[R/K]×3L×6L×3R, a conserved amino acid signature required for interaction of MYBs with R/B-like bHLH transcription factors (Zimmermann et al., 2004), and W×M, a sequence motif that has been shown to be required for cell-to-cell movement of CPC (Kurata et al., 2005).

Trichome formation in *Arabidopsis* is controlled by the interplay of R3 MYBs and several other transcription factors including the WD40-repeat protein TRANSPARENT TESTA GLABRA1 (TTG1; Walker et al., 1999), the R2R3 MYB-type transcription factor GLABRA1 (GL1; Oppenheimer et al., 1991), the bHLH transcription factor GLABRA3 (GL3) or ENHANCER OF GLABRA3 (EGL3; Payne et al., 2000; Zhang et al., 2003), and the homeodomain protein GLABRA2 (GL2; Rerie et al., 1994). TTG1, GL1, and GL3 or EGL3 form an activator complex to induce the expression of *GL2*, a positive regulatory gene of trichome formation (Rerie et al., 1994). The same TTG1–GL3/EGL3–GL1 activator complex can also induce the expression of R3 MYB genes. R3 MYBs can move from a trichome precursor cell to its neighboring cells, and compete with GL1 for binding GL3 or EGL3, thus limiting the formation of the activator complex (Hülskamp et al., 1994; Schellmann et al., 2002; Esch et al., 2003; Schiefelbein, 2003; Pesch and Hülskamp, 2004; Ishida et al., 2008), resulting in inhibition of trichome initiation.

In addition to competing with GL1 for binding GL3, some of the R3 MYBs including TCL1 and TCL2 also directly suppress the expression of *GL1* (Wang et al., 2007; Gan et al., 2011). Not all the R3 MYB genes in *Arabidopsis* are activated by the TTG1–GL3/EGL3–GL1 activator complex (Wang et al., 2008), and *microRNA156* (*MIR156*)-targeted SQUAMOSA PROMOTER BINDING PROTEIN LIKE (SPL) 9 has been shown to activate *TCL1*, *TCL2*, and *TRY* (Yu et al., 2010; Gan et al., 2011). These results indicate that R3 MYBs may use different mechanisms to regulate trichome formation in *Arabidopsis*.

Although single mutants of *Arabidopsis* R3 MYB genes have different phenotypes (Wada et al., 1997, 2002; Schnittger et al., 1999; Schellmann et al., 2002; Wang et al., 2007), over-expression of any of the R3 MYB genes in *Arabidopsis* resulted in glabrous phenotypes. Analysis of double, triple, and higher mutants also revealed that all the seven R3 MYBs function in a highly redundant manner to control trichome formation (Esch et al., 2004; Kirik et al., 2004a, b; Wang et al., 2007, 2008; Tominaga et al., 2008; Wester et al., 2009; Gan et al., 2011).

Functional homologs of some of these transcription factors that regulate trichome formation have been identified in other plants. For example, GaHOX1 from cotton has been identified as a functional homolog of GL2 (Guan et al., 2008) and GaMYB2 as a functional homolog of GL1 (Wang et al., 2004; Guan et al., 2011, 2014). MYB like genes from *Mimulus guttatus* and peach regulate trichome formation (Scoville et al., 2011; Vendramin et al., 2014), and expression of a tomato R3 MYB gene in *Arabidopsis* resulted in glabrous phenotypes (Tominaga-Wada et al., 2013). On the other hand, expression of *Arabidopsis GL3* in *Brassica napus* induced ectopic trichome formation (Gruber et al., 2006). These results suggest that trichome formation in other plant species may be controlled by similar mechanisms as in *Arabidopsis*. However, trichome regulators in plants other than *Arabidopsis* remain largely unidentified.

*Populus trichocarpa* is the first tree whose genome has been fully sequenced (Tuskan et al., 2006), and it is also a good model plant for studies in areas such as wood development, ecological interactions, and other aspects of perennial plants that cannot be studied in the annual model plant *Arabidopsis* (Groover, 2005; Whitham et al., 2006; Jansson and Douglas, 2007). By using the entire amino acid sequence of TCL1 to BLAST search the *P. trichocarpa* protein database, we found there are a total of eight genes in poplar encoding R3 MYB transcription factors, namely *P. trichocarpa TRICHOMELESS1* through *8* (*PtrTCL1–PtrTCL8*). In the study described here, we examined if R3 MYBs from poplar can functionally substitute for *Arabidopsis* R3 MYBs to regulate trichome formation in *Arabidopsis* plants.

## MATERIALS AND METHODS

### IDENTIFICATION OF POPLAR HOMOLOGS OF *Arabidopsis* R3 MYB TRANSCRIPTION FACTOR TCL1

To identify poplar homologs of *Arabidopsis* R3 MYB transcription factors, the entire amino acid sequence of *Arabidopsis* R3 MYB transcription factor TCL1 was used in BLAST searches of the *P. trichocarpa* proteome (www.phytozome.net). The entire amino acid sequences of identified poplar R3 MYB transcription factors were then used in BLAST searches until no more poplar R3 MYBs were identifiable. Full-length amino acid sequences of *Arabidopsis* and poplar R3 MYBs were subjected to phylogenetic analysis using “One Click” mode of Phylogeny (www.phylogeny.fr) with default settings.

### PLANT MATERIALS AND GROWTH CONDITIONS

Poplar xylem tissue from *P. trichocarpa* was collected as described previously (Geraldes et al., 2011; Liu et al., 2013; Wang et al., 2014), and used for RNA isolation and poplar R3 MYB gene cloning. The *tcl1* and *try* mutants, and the *35S:HA-TCL1* transgenic plant were in the Columbia-0 (Col-0) background (Esch et al., 2003; Wang et al., 2007). The *cpc* mutant was in the Ws background (Wada et al., 1997).

Unless specified otherwise, *Arabidopsis* ecotype Col-0 was used for plant transformation. Seedlings used for RNA isolation were obtained by growing sterilized seeds on 1/2 Murashige and Skoog (MS) basal medium with vitamins (Plantmedia) and 1% (w/v) sucrose. Seedlings used for phenotypic analysis were obtained either by growing seeds on 1/2 MS medium or by directly sowing seeds into soil. All plants were grown in growth rooms at 22°C with 14/10 h photoperiod, and light density of approximately 120 μmol m^-^^2^ s^-^^1^.

### RNA ISOLATION AND RT-PCR

Total RNA from poplar samples was isolated using PureLink Plant RNA Reagent (Invitrogen), and cleaned with RNeasy Plant Mini Kit (Qiagen) as described previously (Geraldes et al., 2011; Wang et al., 2014). Total RNA from *Arabidopsis* seedlings was isolated using EasyPure^TM^ Plant RNA Kit (Transgene) according to the manufacturer’s instructions. All RNA samples were treated with RNase-Free DNase set (Qiagen) to eliminate possible DNA contamination.

cDNA was synthesized using 2 μg total RNA by Oligo(dT)-primed reverse transcription using Omniscript RT Kit (Qiagen). Some of the primers used for cloning or examining the expression of corresponding genes have been described previously (Wang et al., 2007, 2008, 2010; Gan et al., 2011), and poplar R3 MYB gene-specific primers are shown in **Table 1**.

**Table 1:** Primers using for poplar R3 MYB gene cloning and expression assays.

Primers	Sequences
*PtrTCL1-Nde1F*	5′-CAACATATGGATAGACGTCGCAGG-3′
*PtrTCL1-Sac1R*	5′-CAAGAGCTCTTAAGAGGTATTAGGAATTAC-3′
*PtrTCL2-Nde1F*	5′-CAACATATGGATAGACGTCGCAAG-3′
*PtrTCL2-Sac1R*	5′-CAAGAGCTCTTAGGAATGACATCTC-3′
*PtrTCL3-Nde1F*	5′-CAACATATGGAGAGTATGAACCGC-3′
*PtrTCL3-Sac1R*	5′-CAAGAGCTCTCAACTAGAAGTCCTAG-3′
*PtrTCL4-Nde1F*	5′-CAACATATGTCCCTTCAATTTCAC-3′
*PtrTCL4-Sac1R*	5′-CAAGAGCTCTTACTGACTTGTAGAGTATC-3′
*PtrTCL5-Nde1F*	5′-CAACATATGGCTGACTTGGATCAC-3′
*PtrTCL5-Sac1R*	5′-CAAGAGCTCTTACTGACTTGTAGAGTATC-3′
*PtrTCL6-Nde1F*	5′-CAACATATGGCTGACTCTGAACATTC-3′
*PtrTCL6-Sac1R*	5′-CAAGAGCTCTCATTCACTTGTAGAGTATC-3′
*PtrTCL7-Nde1F*	5′-CAACATATGGCTGACACTGAACATTC-3′
*PtrTCL7-Sac1R*	5′-CAAGAGCTCTCATTCACTCGTAGAGC-3′
*PtrTCL8-Nde1F*	5′-CAACATATGGCTTGCTCGGGTCAC-3′
*PtrTCL8-Sac1R*	5′-CAAGAGCTCTCATCTTTCCTTTGATGATC-3′

### CONSTRUCTS

To generate HA (human influenza hemagglutinin)- or GD (Gal4 DNA binding domain)-tagged constructs for poplar R3 MYB genes, the full-length, open-reading frames (ORF) of corresponding poplar R3 MYB genes were amplified by RT-PCR using RNA isolated from poplar xylem samples, and the PCR products were then cloned in-frame with an N-terminal HA or GD tag into the *pUC19* vector under the control of the double *35S* enhancer promoter of *CaMV* (Wang et al., 2005).

The *35S:PtrTCL1-GFP* construct was cloned by fusing *PtrTCL1* in frame with GFP (Green fluorescent protein) and then cloned into the *pUC19* vector under the control of the *35S* promoter. The *TCL1p:HA-PtrTCL1*, *TRYp:HA-PtrTCL1*, and *CPCp:HA-PtrTCL1* constructs were cloned by replacing the *35S* promoter in *35S:HA-PtrTCL1* with *TCL1*, *TRY*, and *CPC* promoters, respectively (Wada et al., 1997; Esch et al., 2003; Wang et al., 2007).

For plant transformation, corresponding constructs in the *pUC19* vector were digested with *EcoRI* and subcloned into the binary vector *pPZP211* or *pPZP221* (Hajdukiewicz et al., 1994).

### PLANT TRANSFORMATION AND TRANSGENIC PLANT SELECTION

About five-week-old plants with several mature flowers on the main inflorescence were used for plant transformation. Plants were transformed by using the floral dip method via *Agrobacterium tumefaciens* GV3101 (Clough and Bent, 1998). T1 seeds were geminated on plates containing antibiotics to select transgenic plants. For each construct, more than 70 transgenic lines were obtained. Phenotypes of transgenic plants were examined in the T1 generation and at least five transgenic lines with similar phenotypes were collected. The phenotypes observed were confirmed in the following two to three generations. Expression of corresponding genes in related lines was confirmed by RT-PCR. Homozygous T3 or T4 seeds were used for further experiments, and data from one representative line for each construct are presented.

### PLASMID DNA ISOLATION, PROTOPLAST TRANSFECTION, AND GUS ACTIVITY ASSAY

All reporter and effector plasmids were prepared using the GoldHi EndoFree Plasmid Maxi Kit (Kangwei) according to the manufacturer’s instructions. The procedures for protoplast isolation, transfection, and GUS activity assay have been described previously (Tiwari et al., 2003; Wang et al., 2005, 2008; Wang and Chen, 2008). Briefly, protoplasts were isolated from rosette leaves collected from ~4-week-old *Arabidopsis* plants. Effector and reporter plasmids were co-transfected into protoplasts and incubated at room temperature for 20–22 h under darkness. GUS activities were measured using a Synergy^TM^ HT microplate reader (BioTEK).

### MICROSCOPY

Trichomes were analyzed and photographed using a Motic K microscope equipped with a Canon digital camera. Localization of PtrTCL1-GFP proteins in transgenic plants expressing *PtrTCL1–GFP* under the control of the *35S* promoter was examined under an Olympus FV1000 confocal microscope. Protoplast cells isolated from the *PtrTCL1–GFP* transgenic plants were stained with DAPI and then examined under an Olympus FV1000 microscope.

## RESULTS

### IDENTIFICATION OF R3 MYB TRANSCRIPTION FACTORS IN POPLAR

A total of eight poplar R3 MYB transcription factors were identified, and collectively named as PtrTCL1 to PtrTCL8. Corresponding gene names for the PtrTCLs identified are as follows: *PtrTCL1*, *Potri.002G168900*; *PtrTCL2*, *Potri.014G096300*; *PtrTCL3*, *Potri.015G022000*; *PtrTCL4*, *Potri.007G122800*; *PtrTCL5*, *Potri.017G03700*; *PtrTCL6*, *Potri.011G026300*; *PtrTCL7*, *Potri.004G021300*; and *PtrTCL8*, *Potri.004G015100*.

**FIGURE 1:**
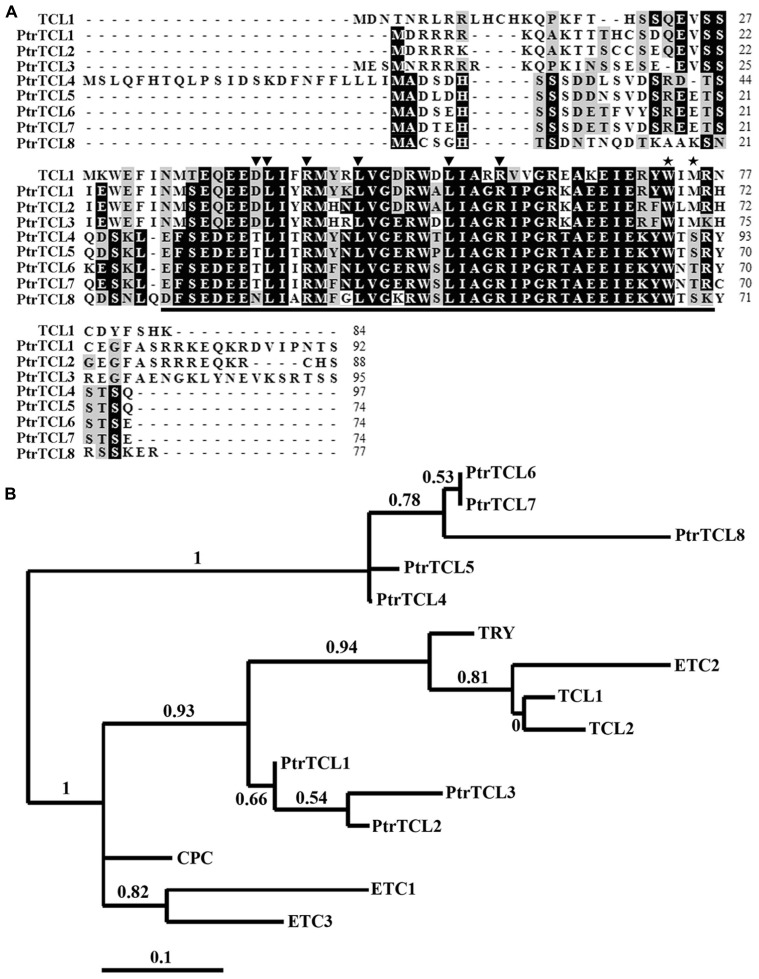
**R3 MYB transcription factors in poplar. (A)** Sequence alignment of TCL1 with eight poplar R3 MYB proteins. Identical amino acids are shaded in black and similar amino acids in gray. The R3 MYB domain is indicated by underline, the amino acid signature [D/E]L×2[R/K]×3L×6L×3R that is required for interacting with R/B-like BHLH transcription factors is indicated by arrowheads, and the amino acids within the MYB domain that are crucial for cell-to-cell movement of CPC are indicated by asterisks. **(B)** Phylogenetic analysis of seven *Arabidopsis* and the eight poplar R3 MYB transcription factors. The entire amino acid sequences were used to generate the phylogenetic tree by using “One Click” mode of Phylogeny (www.phylogeny.fr) with default settings. Branch support values are indicated above or below branches, bar indicates branch length.

Similar to their homologs in *Arabidopsis*, nearly the entire protein of poplar R3 MYBs is made up of the single MYB domain (**Figure 1A**). The amino acid signature [D/E]L×2[R/K]×3L×6L×3R, that is required for the interaction with R/B-like bHLH transcription factors (Zimmermann et al., 2004), is fully conserved in all seven R3 MYB transcription factors in *Arabidopsis* (Wang et al., 2007, 2008; Gan et al., 2011), but is found in only three poplar R3 MYBs including PtrTCL1, PtrTCL2, and PtrTCL3 (**Figure 1A**). In the other four poplar R3 MYBs, D/E, the first amino acid in the amino acid signature is replaced by T/N (**Figure 1A**). Similarly, the amino acid motif W×M, that has been shown to be required for the cell-to-cell movement of CPC (Kurata et al., 2005), is conserved in all seven *Arabidopsis* R3 MYBs (Wang et al., 2007, 2008; Gan et al., 2011), but is found in only PtrTCL1, PtrTCL2, and PtrTCL3 (**Figure 1A**). In the other four poplar R3 MYBs, the M in the motif is replaced by S/T (**Figure 1A**).

Phylogenetic analysis using full-length protein sequences of poplar R3 MYBs and TCL1 showed that the clade of PtrTCL1–PtrTCL3 is most closely related to the clade of TCL1, TCL2, ETC2 and TRY (**Figure 1B**). Together, PtrTCL1–PtrTCL3 and seven *Arabidopsis* R3 MYBs formed one subgroup, and PtrTCL4–PtrTCL8 formed another subgroup.

### PtrTCLs INTERACT WITH GL3

We have previously demonstrated that TRY, CPC, ETC1, ETC2, TCL1, TCL2, and ETC3 interact with GL3 in plant cells (Wang et al., 2008; Gan et al., 2011), supporting the proposal that R3 MYBs control trichome formation in *Arabidopsis* by competing with GL1 for binding GL3, and thus eliminating the formation of TTG1–GL3–GL1 activator complex. Considering that only three of the identified poplar R3 MYBs have the fully conserved amino acid signature that is required for the interaction with R/B-like bHLH transcription factors, we tested if PtrTCLs interact with GL3 in plant cells.

**FIGURE 2:**
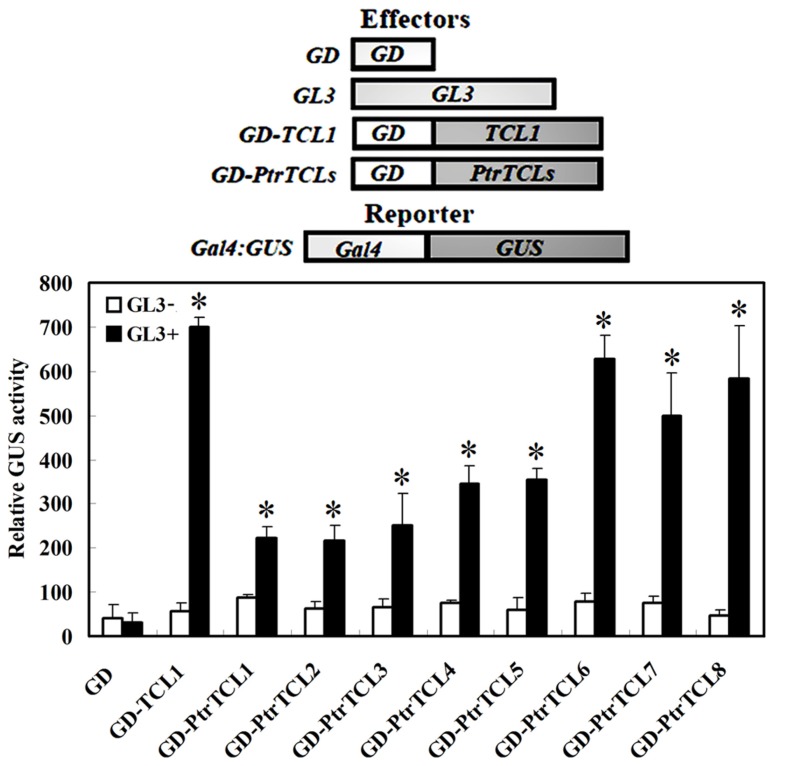
**Poplar R3 MYBs interact with GL3 in plant cells**. Reporter gene and effector gene plasmids were co-transfected into protoplasts isolated from *Arabidopsis* rosette leaves. Protoplasts were incubated in darkness for 20–22 h after transfection, and then GUS activity was measured. Data represent the mean±SD of three replicates. Reporter and effector constructs are diagrammed at the top of the figure. *significantly different from the absence of GL3 (GL3-; *P* < 0.001).

*Arabidopsis* protoplast transfection assays were used to test the interaction between PtrTCLs and GL3. Plasmids of the reporter gene *Gal4-GUS*, together with the effector genes *GL3* and GD fused PtrTCLs (*GD–PtrTCLs*; **Figure 2**), were co-transfected into *Arabidopsis* protoplasts. *GD* and *GD–TCL1* were used as negative and positive controls, respectively. As expected, neither GD nor GD–TCL1 activated the reporter gene in the absence of GL3. In the presence of GL3, GD–TCL1 but not GD activated the reporter gene. Similarly, none of the eight poplar R3 MYBs activated the reporter gene in the absence of GL3; however, all of them activated the reporter gene in the presence of GL3 (**Figure 2**).

### PtrTCLs NEGATIVELY REGULATE TRICHOME FORMATION WHEN EXPRESSED IN *Arabidopsis*

**FIGURE 3:**
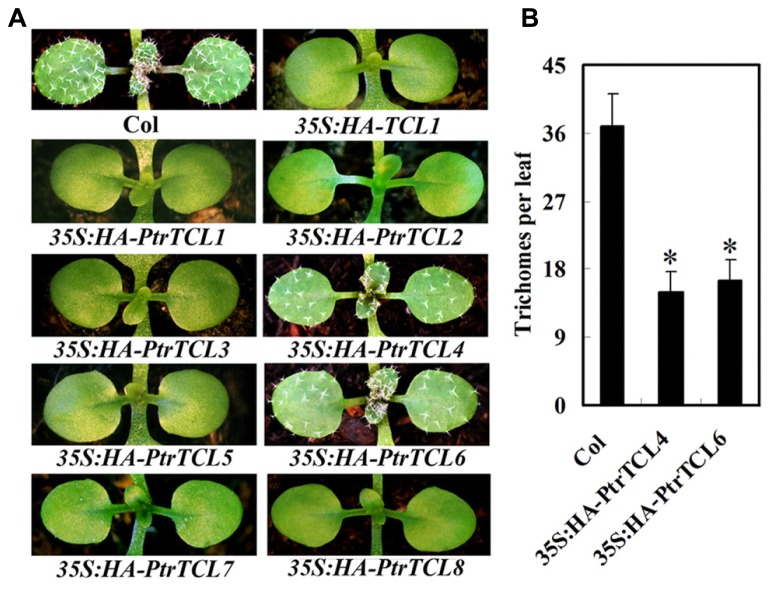
**Poplar R3 MYBs negatively regulate trichome formation in *Arabidopsis.* (A)** Trichome phenotypes in *Arabidopsis* plants expressing poplar R3 MYB genes. All transgenic lines are in Col background. Pictures were taken from 2-week-old, soil-grown seedlings. **(B)** Trichome density on the first two rosettle leaves of *Arabidopsis* plants expressing *PtrTCL4* and *PtrTCL6*. Data represent the mean±SD of 18 plants. *significantly different from Col wild-type plants (*P* < 0.001).

**FIGURE 4:**
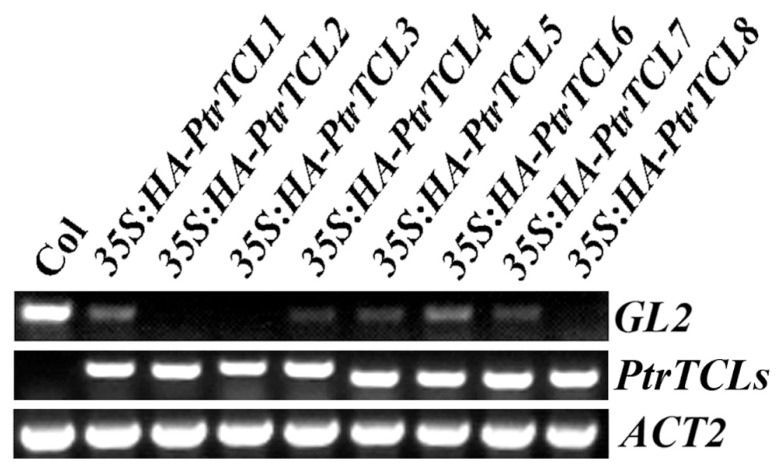
**Expression of *GL2* and poplar R3 MYB genes in transgenic *Arabidopsis* plants**. RNA was isolated from Col wild-type and transgenic *Arabidopsis* seedlings, RT-PCR was used to examine the expression of *GL2* and polar R3 MYB genes. The expression of *ACTIN2* (*ACT2*) was used as a control.

Having shown that all the eight poplar R3 MYBs interact with GL3 in plant cells, we further analyzed if PtrTCLs regulate trichome formation by generating transgenic *Arabidopsis* plants expressing HA-tagged PtrTCLs under the control of *35S* promoter (*35S:HA-PtrTCLs*). As shown in **Figure 3A**, expression of *PtrTCL1*, *PtrTCL2*, *PtrTCL3*, *PtrTCL5*, *PtrTCL7* and *PtrTCL8* in *Arabidopsis* resulted in glabrous phenotypes, similar to the phenotype observed in plants over-expressing *TCL1* (Wang et al., 2007). In contrast, transgenic plants expressing *PtrTCL4* and *PtrTCL6* had greatly reduced number of trichomes on rosette leaves, but none of the transgenic plants obtained showed glabrous phenotypes (**Figure 3**). RT-PCR analysis showed that poplar R3 MYB genes were highly expressed in their corresponding transgenic lines (**Figure 4**), indicating that the phenotypes observed in *PtrTCL4* and *PtrTCL6* transgenic plants were not due to relative lower expression levels of the corresponding genes.

*GL2* is one of the target genes of the activator complex TTG1–GL3/EGL3–GL1, and it positively regulates trichome formation in *Arabidopsis*. Because binding of PtrTCLs to GL3 indicated that expression of *PtrTCLs* in *Arabidopsis* resulted in the inhibition of the formation of TTG1–GL3/EGL3–GL1 activator complex, we examined the expression of *GL2* in *Arabidopsis* transgenic plants expressing poplar R3 MYB genes. As shown in **Figure 4**, expression of *GL2* was dramatically reduced in transgenic plants expressing any of the poplar R3 MYB genes.

### SUBCELLULAR LOCALIZATION OF PtrTCL1

**FIGURE 5:**
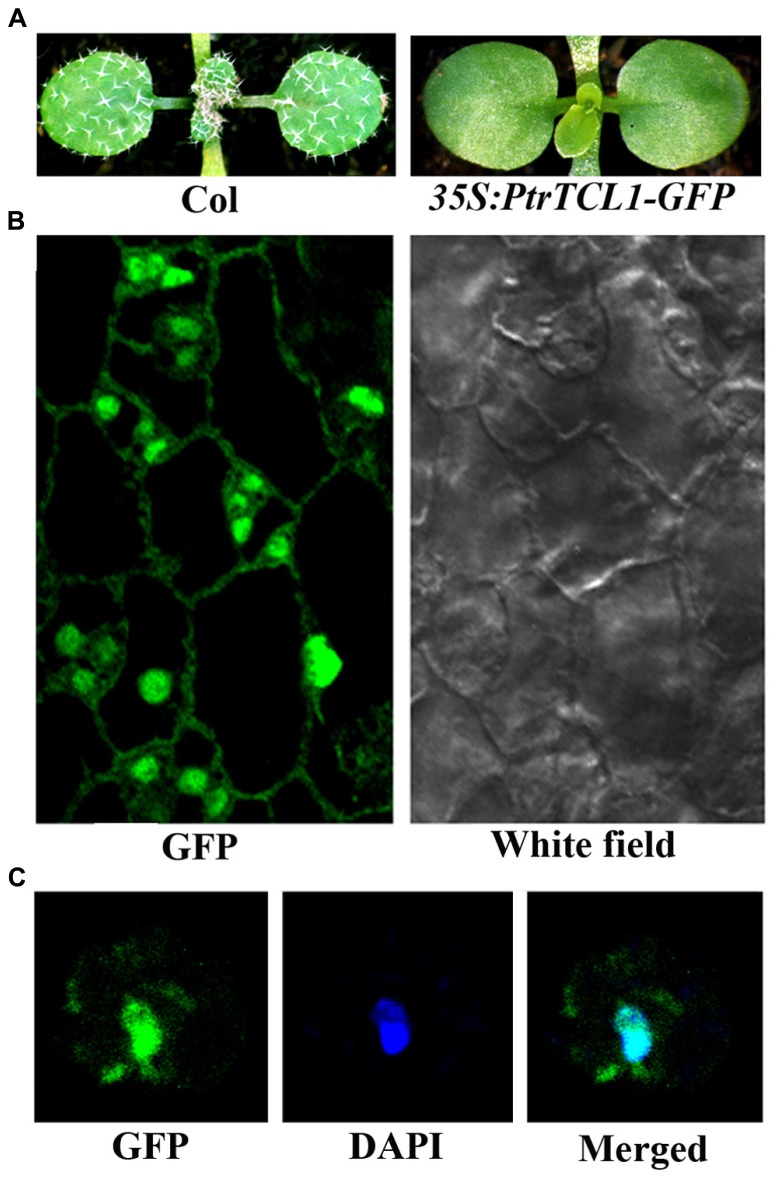
**Subcellular localization of PtrTCL1. (A)** Expression of *PtrTCL1-GFP* under the control of *35S* promoter in *Arabidopsis* resulted in a glabrous phenotype. Pictures were taken from 2-week-old, soil-grown seedlings. **(B)** GFP fluorescence in the epidermal cells of the rosettle leaves in a 2-week-old transgenic plant expressing *PtrTCL1-GFP* under the control of the *35S* promoter. Left panel: GFP channel, right panel: white field image. **(C)** GFP fluorescence in a protoplast cell isolated from transgenic plant expressing *PtrTCL1-GFP* under the control of the *35S* promoter. Left panel: GFP channel; Middle panel: DAPI channel; right panel: merged.

Among the eight poplar R3 MYB transcription factors, PtrTCL1 has highest amino acid similarity with TCL1, and phylogenetic analysis also showed that the clade of PtrTCL1–PtrTCL3 is most closely related to TCL1 (**Figure 1**). We wanted to further explore the functions of poplar R3 MYB transcription factors in the regulation of trichome formation in *Arabidopsis* by taking PtrTCL1 as an example. We first examined the subcellular localization of the PtrTCL1 protein by generating transgenic plants expressing *PtrTCL1–GFP* under the control of the *35S* promoter. As shown in **Figure 5A**, expression of *PtrTCL1–GFP* in *Arabidopsis* resulted in a glabrous phenotype, a phenotype similar to transgenic plants expressing *PtrTCL1* (**Figure 3**). This indicated that the PtrTCL1–GFP fusion protein is likely functional. By examining the transgenic plants obtained, we found that GFP florescence was mainly observed in the nucleus of epidermal cells (**Figure 5B**); examination of DAPI stained protoplast cells isolated from the *PtrTCL1–GFP* transgenic plants confirmed that the GFP was mainly observed in the nucleus (**Figure 5C**), indicating that PtrTCL1 is a nuclear localized protein.

### PtrTCL1 ALMOST COMPLETELY RESCUED *tcl*1 MUTANT TRICHOME PHENOTYPES

Previously, we identified *TCL1* as a major R3 MYB transcription factor that regulates trichome formation on the inflorescences and pedicels, and we showed that knock-out of *TCL1* resulted in ectopic trichome formation on the inflorescence stems and pedicels. In addition, we showed that over-expression of *TCL1* in *Arabidopsis* resulted in a glabrous phenotype (Wang et al., 2007). Here, we showed that PtrTCL1, that is in the most closely related clade to TCL1 (**Figure 1**), resulted in a glabrous phenotype when over-expressed in *Arabidopsis* (**Figure 3**). Therefore, we wanted to further examine if PtrTCL1 is the functional equivalent of TCL1 by testing if *PtrTCL1* could rescue the *tcl1* mutant phenotype when expressed under the control of *TCL1* native promoter.

**FIGURE 6:**
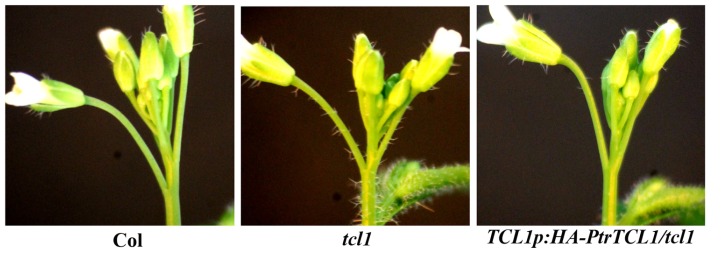
**Rescue of *tcl1* phenotype by *PtrTCL1***. Trichome formation on inflorescences in Col, *tcl1* and *TCL1p:HA-PtrTCL1*/*tcl1-1* plants. Pictures were taken from 5-week-old, soil-grown plants.

Transgenic plants were generated in a *tcl1* background to express *PtrTCL1* under the control of the *TCL1* native promoter (*TCL1p:HA-PtrTCL1/tcl1*). Previously, we showed that expression of *TCL1–GFP* under the control of the *TCL1* promoter fully rescued the *tcl1* trichome phenotype (Wang et al., 2007), indicating that the *TCL1* promoter used is functional in *Arabidopsis*. As shown in **Figure 6**, expression of *PtrTCL1* under the control of *TCL1* native promoter almost completely rescued *tcl1* mutant phenotype, i.e., *tcl1* mutant forms ectopic trichomes in both stem internodes above the first flower and pedicels, while *TCL1p:HA-PtrTCL1/tcl1* plants do not have trichomes on stem internodes after the first flowers, but have a few trichomes on first pedicel only. These results indicate that PtrTCL1 is likely the functional equivalent of TCL1 in controlling trichome formation on the inflorescence stems and pedicels.

**FIGURE 7:**
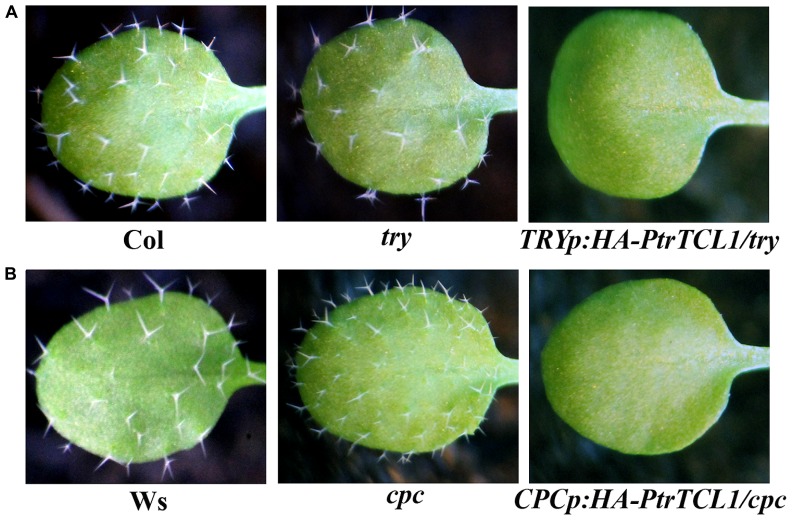
**Expression of *PtrTCL1* in *try* and *cpc* resulted in glabrous phenotypes. (A)** Expression of *PtrTCL1* under the control of *TRY* native promoter in the *try* mutant background resulted in glabrous phenotype. **(B)** Expression of *PtrTCL1* under the control of *CPC* native promoter in the *cpc* mutant background resulted in glabrous phenotype. Pictures were taken from 2-week-old, soil-grown seedlings.

In addition to *tcl1*, single mutants *try* and *cpc* showed trichome phenotypes; *try* mutants have trichome clusters, and *cpc* mutants have increased numbers of trichome on leaves (Wada et al., 1997; Schnittger et al., 1999), so we also examined if PtrTCL1 is functionally equivalent to *TRY* or *CPC*. Transgenic plants were generated to express *PtrTCL1* under the control of *TRY* native promoter (*TRYp:HA-PtrTCL1/try*) in the *try* background, and under the control of *CPC* native promoter (*CPCp:HA-PtrTCL1/cpc*) in the *cpc* background. As shown in **Figure 7**, expression of *PtrTCL1* under the control of *TRY* native promoter in a *try* mutant background, or under the control of *CPC* native promoter in a *cpc* mutant background resulted in glabrous phenotypes.

## DISCUSSION

In this study we identified poplar homologs of *Arabidopsis* R3 MYB transcription factors, and analyzed their function in trichome formation in *Arabidopsis*. We showed that expression of any of the eight poplar R3 MYB genes under the control of the *35S* promoter in *Arabidopsis* resulted in either a glabrous phenotype or a great reduction in trichome numbers (**Figure 3**). These results suggest that poplar R3 MYBs act as negative regulators for trichome formation and may have overlapping functions, similar to their *Arabidopsis* R3 MYB homologs.

Among the eight poplar R3 MYB transcription factors, PtrTCL1 is in the most closely related clade to TCL1 (**Figure 1B**), is localized in the nucleus (**Figure 5**), and expression of *PtrTCL1* under the control of the *TCL1* promoter in the *tcl1* mutant background (*TCL1p:HA-PtrTCL1/tcl1*) almost fully restored the trichome phenotype of the *tcl1* mutant (**Figure 6**). Expression of *PtrTCL1* under the control of the *TRY* native promoter in the *try* background (*TRYp:HA-PtrTCL1/try*), or under the control of the *CPC* native promoter in the *cpc* background (*CPCp:HA-PtrTCL1/cpc*) resulted in glabrous phenotypes (**Figure 7**), suggesting that PtrTCL1 may be the functional equivalent of TCL1, rather than TRY and CPC. It should be noted that ETC2 and TCL2 are also in the clade that is sister to the clade of PtrTCL1–PtrTCL3; however, because no mutant available for *TCL2* and *etc2* mutant does not have any trichome phenotype, we could not test whether PtrTCL1 might be functionally equivalent to TCL2 or ETC2.

In *Arabidopsis*, R3 MYBs inhibit trichome formation by competing with GL1 for binding of GL3 or EGL3, thus inhibiting the formation of the TTG1–GL3/EGL3–GL1 activator complex (Schellmann et al., 2002; Esch et al., 2003; Schiefelbein, 2003; Pesch and Hülskamp, 2004; Ishida et al., 2008). In accordance with these results, all seven *Arabidopsis* R3 MYBs have the conserved [D/E]L×2[R/K]×3L×6L×3R amino acid signature that is required for interaction with R/B-like bHLH transcription factors (Zimmermann et al., 2004), and protoplast transfection assays showed that they all interact with GL3 in plant cells (Wang et al., 2008; Gan et al., 2011). Sequence alignment results showed that only three of the poplar R3 MYBs, namely PtrTCL1, PtrTCL2, and PtrTCL3, have the fully conserved amino acid signature required for interacting with bHLH transcription factors (**Figure 1A**). When tested in protoplasts, however, all the eight poplar R3 MYBs interacted with GL3 (**Figure 2**), and the interaction with GL3 may be stronger for those R3 MYBs without the fully conserved amino acid signature as judged by the GUS activities (**Figure 2**). Our results indicate that either the conserved amino acid signature is not required for the interaction of poplar R3 MYBs with GL3, or a single-amino-acid substitution (D/E > T/N) in poplar R3 MYBs does not affect their interaction with GL3. It is also possible that previous designations of conserved residues may be biased due to a smaller set of proteins analyzed. Furthermore, interaction of poplar R3 MYBs with GL3 in plant cells suggests that poplar R3 MYBs can block the formation of the activator complex required for trichome formation. In accordance with this, RT-PCR results showed that expression of *GL2* is dramatically reduced in the transgenic plants expressing poplar R3 MYB genes (**Figure 4**). Since our previous results showed that R3 MYBs in *Arabidopsis* may regulate trichome formation in a GL2 independent manner (Wang et al., 2010), we cannot rule out the possibility that poplar R3 MYBs may also regulate trichome formation through other mechanisms.

## Conflict of Interest Statement

The authors declare that the research was conducted in the absence of any commercial or financial relationships that could be construed as a potential conflict of interest.
